# High Performance Polymeric Fabry-Pérot Microcavities for Sensing and Lasing Applications

**DOI:** 10.3390/polym17182496

**Published:** 2025-09-16

**Authors:** Genni Testa, Vito Coviello, Gianluca Persichetti, Romeo Bernini

**Affiliations:** Institute for Electromagnetic Sensing of the Environment (IREA), National Research Council (CNR), Via Diocleziano 328, 80124 Naples, Italy; coviello.v@irea.cnr.it (V.C.); persichetti.g@irea.cnr.it (G.P.); bernini.r@irea.cnr.it (R.B.)

**Keywords:** Fabry-Pérot, optical microcavity, optofluidic microlaser, polymer photonics

## Abstract

We present the design, fabrication, and optical characterization of fully polymer-based high performance Fabry-Pérot microcavities for sensing and lasing applications. Two microcavity types (Cavity A and B) were realized using polymeric Distributed Bragg Reflector (DBR) films offering distinct spectral properties. Cavity A achieved a high quality factor (Q ≈ 2.15 × 10^5^), demonstrating excellent sensitivity for bulk refractive index sensing with an ultrahigh figure of merit of 5.89 × 10^4^ and a theoretical detection limit down 3.4 × 10^−7^ RIU. Cavity B was optimized for lasing applications. When filled with a Rhodamine B dye solution, it exhibited clear lasing action with a low threshold (1.83 μJ/mm^2^) and resonant peaks consistent with its free spectral range. These results highlight the potential of cost-effective polymeric cavities for disposable photonic sensor platforms and integrated biolaser devices.

## 1. Introduction

Optical microcavities have long been a fundamental component in photonic systems, enabling precise control over light-matter interactions for applications ranging from quantum optics to sensing. By confining light in small volumes, these structures significantly enhance light–matter interactions, enabling effects such as strong coupling, Purcell enhancement, and high-quality (Q) factor resonances [1]. As a result, microcavities have played a significant role in advancing technologies like low-threshold lasers and highly sensitive optical sensors on small scale [2,3,4] where small changes in the cavity conditions can produce measurable shifts in the cavity optical response.

Several types of microcavities have been developed, including whispering gallery mode (WGM) resonators, photonic crystal cavities, and Fabry-Pérot (FP) configurations [1].

Among various cavity designs, the FP configuration remains one of the most widely implemented on the micron scale due to its simplicity in both the fabrication of the mirrors and the cavity itself [5,6]. Fabry-Pérot resonators are a class of open-access optical cavities consisting of two mirrors set parallel to each other. Thanks to their open geometry, these cavities are readily integrable into microfluidic environments for sensing applications [7]. In particular, configurations with planar and parallel mirrors (P-P) are attractive due to their straightforward and cost-effective fabrication compared to plano-concave FP microcavity (P-C). Howeverthey tend to be more susceptible to mechanical and environmental instabilities compared to monolithic or curved-mirror designs.

Due to their straightforward architecture, P-P FP cavities have been successfully assembled by aligning two mirrors, such as metallic-coated [8,9] or polymer-based [10] flat substrates facing each other.

However, the fabrication of optical microcavities has predominantly been based on rigid inorganic materials, such as silicon, gallium arsenide, or silica [2,11,12,13], which are the best choice due to their excellent optical properties. Yet, these materials are expensive to process and often incompatible with flexible or disposable substrates. Achieving high-Q and high-finesse resonators requires mirrors with extremely high reflectivity, which typically involves the fabrication of dielectric distributed Bragg reflectors using deposition processes and, for concave mirrors, advanced fabrication techniques like lithographic etching, laser micromachining, or focused ion beam milling. Notably, recent progress in lithographically aligned silicon mirrors has led to FP cavities with finesse exceeding 5 × 10^5^ [13,14].

In contrast, polymer-based materials offer an attractive alternative due to their low cost, mechanical flexibility, and compatibility with high-throughput fabrication techniques. Methods such as spin-coating, soft lithography, extrusion, and roll-to-roll printing allow for large-area processing of polymeric structures on diverse substrates [15].

Polymeric mirrors, also known as polymeric DBRs, are typically fabricated by stacking multiple layers of polymer materials with alternating refractive indices [16,17,18].

While early polymeric mirrors faced challenges in achieving comparable reflectivity to their inorganic counterparts, recent advancements in material engineering have significantly improved their performance, making them a viable alternative for high-performance photonic systems.

In recent years, companies such as 3M and Toray have developed advanced coextrusion and orientation technologies that allow precise control over both layer thickness and refractive index at the nanometer scale. Thanks to these innovations, a wide range of thin multilayer films with customized spectral properties are now commercially available.

These polymeric mirrors offer significant potential for the development of optical microcavities that are not only highly efficient but also low-cost and suitable for integration into disposable sensing platforms [19].

In this work, we design and characterize fully polymer-based optical microcavities with different spectral properties, demonstrating their promising high optical performance in different applications. These cavities are fabricated using low-cost cold lamination techniques and integrated microfluidics to control liquid injection and implement flow-through measurements.

We have successfully developed cavities with high quality factors able to perform high-sensitivity refractive index sensing with strong improvements respect to our previous device [19], but also suitable to sustain low threshold lasing action. These characteristics make these cavities promising candidates for cost-effective biosensors devices based on either label-free detection or lasing effect, such as biolaser-based sensing platform [20,21,22]. The results establish polymer microcavities as versatile platforms for disposable photonic sensors, combining the advantages of mechanical flexibility, scalable fabrication, and competitive optical performance.

## 2. Methods

### 2.1. Design and Fabrication of Cavity A and Cavity B

We have fabricated two types of cavities, which, besides having some slight and not-substantially differences in the fabrication process, differ primarily in the composition of their mirrors. In particular, we used two cost-effective types of polymeric DBR films, commercially available from 3M Company: DF-Blaze (DFB) and DF2000MA (DF2) films.

These DBR films exhibit different spectral reflectivity profiles, which influence their suitability for different optical applications.

DF2 films consist of more than 600 layers with alternating layers of Polyethylene Naphthalate (PEN n_2_ = 1.75) and acrylic polymer (PMMA n_1_ = 1.49) with a total thickness of 66 µm and a high reflectance (R > 99%) over a spectral bandwidth that ranges from 400 nm up to 900 nm.

DFB films consist of about 250 layers with alternating layers of polyester (PET n_2_ = 1.60) and acrylic polymer (PMMA n_1_ = 1.49) with a total thickness of 33 µm and a high reflectance (R > 96%) over a spectral bandwidth that ranges from 570 nm up to 700 nm.

In one cavity, DF2 were used to realize both mirrors (Cavity A). In the second device (Cavity B), instead, the mirrors were made using DFB.

Cavities A and B were designed with distinct optical properties to meet different application requirements, which is reflected in the choice of DBR films used in their construction. Cavity A employs mirrors with a broadband reflectance range, making it suitable for applications that require wide spectral coverage. In contrast, Cavity B utilizes DBRs with a narrower reflectance band, optimized for more spectrally selective tasks.

Cavity A, with its broader spectral response, is particularly advantageous for sensing applications. The wide reflectance range of the DF2 mirrors allows Cavity A to operate across the visible and near-infrared region up to 900 nm, enabling the detection of resonance wavelength shift over a wide range of refractive indices or analyte absorption features. This broadband behavior enhances its versatility as a sensing platform, especially in applications where the optical response of the analyte is not limited to a narrow spectral window.

**Figure 1:**
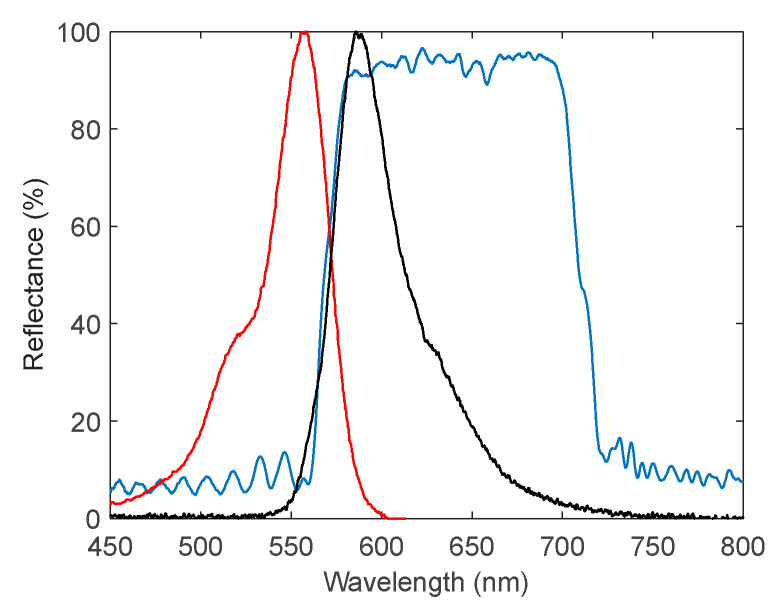
Measured reflectance of the DFB film (blue curve) used for Cavity B. For comparison, the normalized measured absorption (red) and emission spectrum (black) of the Rhodamine B dye solution in water are also shown.

Cavity B, on the other hand, was preferred for lasing due to its narrow reflectance band, which offers inherent spectral filtering. As reported in Figure 1, the spectral properties of the DFB film match very well the absorption/emission properties of the absorption and emission spectra of Rhodamine B, which can hence be chosen as a gain medium. This spectral overlap enables efficient excitation of the gain medium by the pump laser during its single-pass through the cavity, while the emitted fluorescence remains confined within the resonant structure, leading to optical feedback and the onset of lasing.

**Figure 2:**
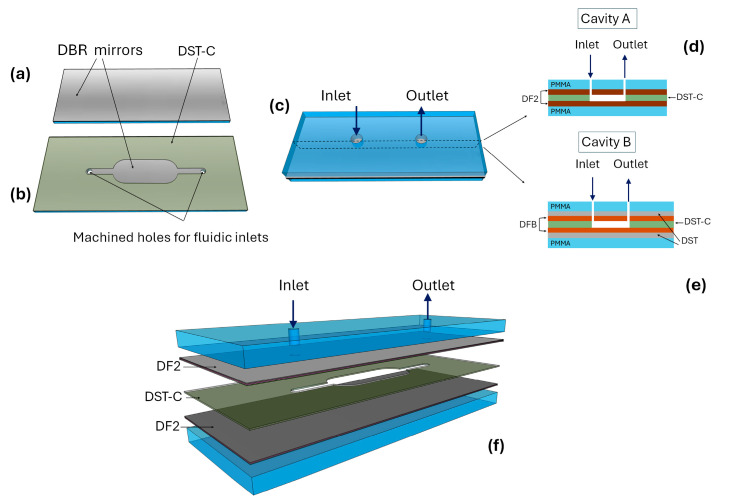
(**a**) PMMA substrate with laminated DBR mirror and (**b**) PMMA substrate with laminated DBR mirror, DST-C layer and fabricated fluidic holes. (**c**) Schematic of the assembled device with transverse section of (**d**) Cavity A and (**e**) Cavity B. (**f**) Exploded view drawing of Cavity A.

Fabrication steps of the cavities are schematized in Figure 2. The cavities consist of two halves of PMMA substrates. The first step involves laminating the top and bottom DBR mirrors onto both PMMA substrates (Figure 2a,b). After that, a double-sided tape (DST-C) is laminated onto one of the PMMA substrates (Figure 2b). Each layer is carefully applied using a laminating roller, ensuring a bubble-free bond at every stage.

The DST-C is pre-processed to include a precisely defined cavity structure before bonding. This pre-formed cavity assures that, once the two halves are joined, they form an optically resonant space with controlled dimensions. Next, we created two holes for fluid flow. Two 0.5 mm diameter holes are drilled by computerized numerical control (CNC) micro milling machine into the top substrate, passing through the mirror (Figure 2b). These holes serve as the inlet and outlet ports for fluidic connections, enabling the cavity to be fully filled with liquid

The final step involves bonding the two processed PMMA substrates using DST-C (Figure 2c). This ensures that when the mirrors are joined, the cavity is perfectly formed and closed. The cavity length is determined by the thickness of the DST-C, which provides both adhesion and alignment accuracy. The lengths of cavity A and B were 40 µm and 50 µm, respectively.

The integration of fluidic connections enables precise injection of sample fluid into the cavity (Figure 2d,e). The fluid is injected through the inlet hole. It flows into and completely fills the microfluidic channel that is machined into the DST-C spacer. This channel defines the optical cavity volume. The fluid is confined horizontally by the channel walls and vertically by the two polymer halves that form the device structure. This design fully implements a flow-through approach, ensuring efficient and controlled sample transport through the cavity. The optimized flow path reduces sample waste while enabling rapid replenishment, critical for dynamic studies such as real-time monitoring or sequential analysis.

The main difference between the fabrication procedure of DF2-based (Cavity A) and DFB-based (Cavity B) cavities lies in their mirror structures. A schematic transverse section of Cavity A and Cavity B are shown in Figure 2d and Figure 2e, respectively. DF2 mirrors are tape-like, meaning they come with an integrated adhesive layer, allowing direct lamination onto the PMMA substrate. In contrast, DFB mirrors are non-adhesive films, requiring an additional double-sided tape (DST)for bonding. In this case, we employed a 50 µm-thick optically transparent tape to ensure proper attachment while maintaining optical transparency and structural integrity.

The integrity of the cavity is maintained by a robust bonding process. The cavity is formed by pressing together the two polymer halves, which have the mirrors laminated on their inner surfaces. These two halves are permanently bonded together using DST-C. The cavity volume is machined into this tape, ensuring a leak-proof seal that prevents any fluid leakage from the cavity.

### 2.2. Experimental Set-Up

**Figure 3:**
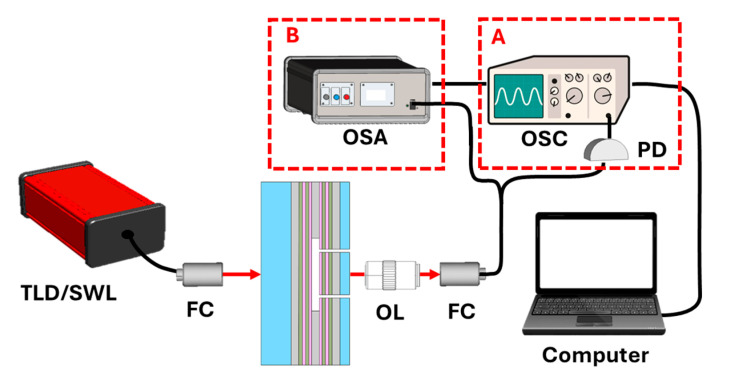
Schematic of the two experimental set-up configurations for characterization of Cavity A and B. They differ only in the excitation source and the detection equipment, the latter indicated by the dashed line in boxes A and B (based on the cavity names).

The optical properties of Cavity A were characterized using a tunable laser diode (TLD) with an emission wavelength range of 765–781 nm (TLB 6700, New Focus), Irvine, CA, USA. The laser output was coupled into a single-mode (SM), polarization-maintaining GRIN fiber collimator (FC). The transmitted signal was collected through a 5× objective lens (OL) and sent to a photodiode (PD) connected to an oscilloscope (OSC) for spectral measurements (Figure 3, configuration A). The OSC records the optical signal received from the PD as a function of time. Since this time is related to the scan wavelength of the tunable source, the optical spectrum can be reconstructed from the OSC signal. To achieve precise optical alignment, we used micrometer-resolution kinematic mirror mounts and translation stages.

To assess its sensing capabilities, Cavity A was tested as a bulk refractive index sensor. Glycerol-water solutions of varying concentrations were prepared and characterized using an Abbe refractometer before being introduced into the cavity for sensitivity testing.

The measured refractive index values ranged from 1.3305 to 1.33259. The optical performance of Cavity B was characterized in transmission mode using a 5 nm band passed supercontinuum white light optical source (SWL) and an optical spectrum analyzer (OSA, resolution 10 pm at 630 nm) (Figure 3, configuration B). During the measurement, the cavity was filled with water through the integrated microfluidic channels. The diameter of the incident light beam was 1 mm.

**Figure 4:**
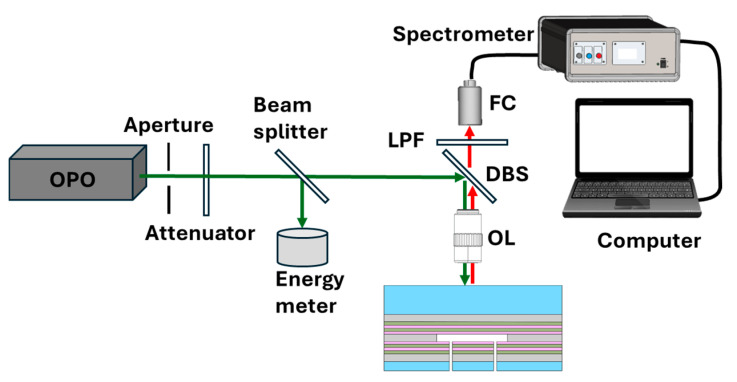
Schematic of the experimental set-up for lasing characterization of Cavity B.

The experimental set-up for lasing characterization is depicted in Figure 4. Measurements were performed in reflection mode. A laser beam, with a wavelength of 557 nm, pulse duration of 8 ns and a repetition rate of 10 Hz, emitted by a pulsed optical parametric oscillator (OPO) (GWU OPO primoScan, Erftstadt, Germany) pumped by a Nd:YAG lasers (Quantel Q-smart, Les Ulis Cedex, France), is normally incident onto the FP microcavity through the front planar mirror with a focal spot size of about 160 μm. The pump energy can be varied using a polarization-based laser attenuator. The lasing generated by the excitation of the gain medium is filtered out by dichroic beam splitter and a long-pass filter (LPF) and finally collected by an optical fiber and delivered to a spectrometer (resolution 0.6 nm).

For laser generation, the microcavity was filled with an aqueous Rhodamine B dye solution (1 mM) serving as the gain medium.

## 3. Results

**Figure 5:**
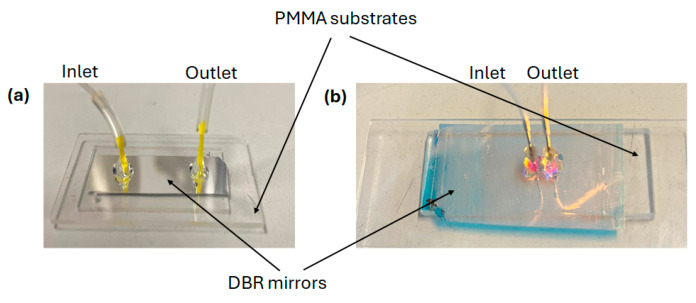
Photographs of fabricated (**a**) Cavity A and (**b**) Cavity B.

In Figure 5 are shown the photographs of the fabricated devices, featuring two distinct cavity structures (A and B) designed for their respective optical functionality.

### 3.1. Cavity A—Refractive Index Sensing

**Figure 6:**
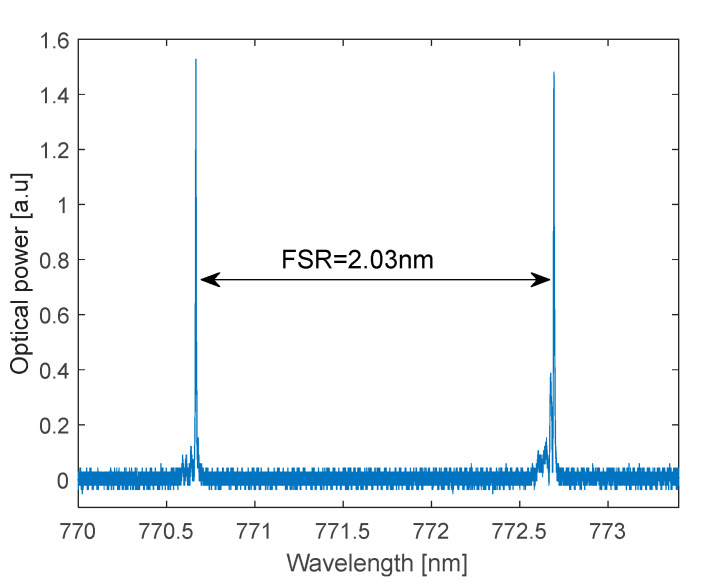
Measured spectrum transmitted by Cavity A filled with water.

**Figure 7:**
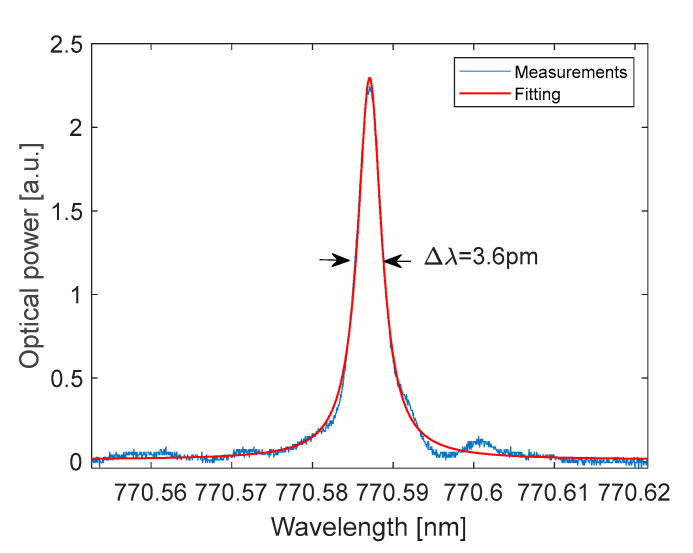
Optical resonance and Lorentzian fit at around λ = 770.59 nm.

Figure 6 shows the measured transmission spectrum of Cavity A. During the measurement, the cavity was filled with water through the integrated microfluidic channels. Distinct optical resonance peaks arise from constructive interference due to multiple reflections between the cavity mirrors (Figure 6). A detailed view of one optical resonance is shown in Figure 7, where the resonance has been fitted with a Lorentzian curve. The full width at half maximum (FWHM) of the resonances is approximately Δλ ≈ 3.6 pm, corresponding to a Q-factor of 2.15 × 10^5^. The measured free spectral range (FSR) is 2.03 nm. We measured a cavity finesse F = FSR/Δλ = 554.

The measured Q-factor demonstrates the high optical quality of the fabricated cavity, which is competitive with state-of-the-art optofluidic resonators [22]. This value reflects low optical losses and efficient light confinement within the cavity, making it suitable for high-resolution label-free detection in microfluidic environments.

**Figure 8:**
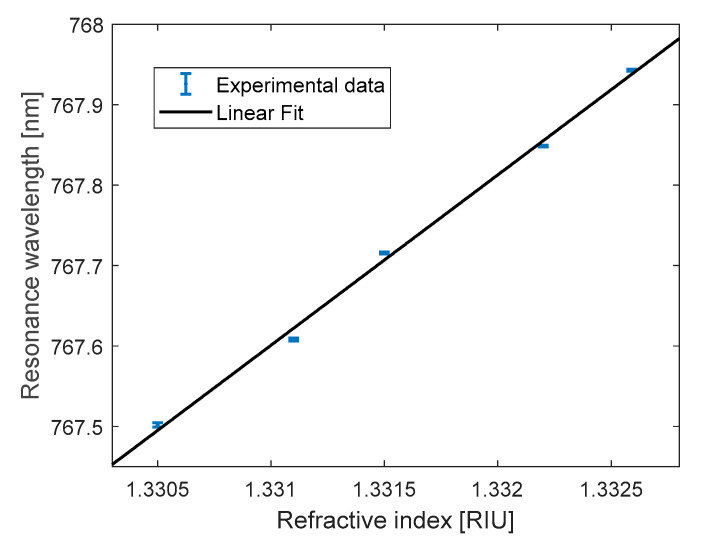
Resonant wavelengths versus the cavity RI.

By injecting liquids of varying refractive indices (RI) into the cavity through the integrated microfluidics, we measured resonant wavelength shifts in the transmission spectrum. These shifts vary with the RI of the analyte, enabling sensitivity calibration. Experimental results and statistical analysis are presented in Figure 8.

Each data point in Figure 8 corresponds to the mean resonant wavelength derived from fifteen measurements, with error bars indicating the standard deviation. The bulk refractive index sensitivity was determined from the slope of the linear regression fit to the resonant wavelength versus refractive index data, yielding a sensitivity of 212 ± 7 nm/RIU (R^2^ = 0.9969). The observed uncertainty arises primarily from (i) environment temperature fluctuation and (ii) the measurement accuracy of the Abbe refractometer available in our laboratory (±2.5 × 10^−4^ RIU resolution). The refractive index limit of detection (LOD), defined as the smallest detectable refractive index variation, depends on both the sensitivity (S) and the spectral resolution (δλmin) following the relation LOD = δλmin/S. Typically, δλmin ranges between Δλ/50 and Δλ/100 and is influenced by noise sources [23]. Under the assumption of δλmin = Δλ/50 (representing the worst-case scenario), the bulk sensitivity measurement suggests a theoretical refractive index LOD of 3.4 × 10^−7^ RIU. By considering that the mean standard deviation of the peak wavelengths reported in Figure 7 is equal to 2.2 pm, the LOD with this experimental set-up is limited to 1.04 × 10^−5^ RIU, mainly due to the absence of thermal stabilization of the system.

### 3.2. Cavity B—Active Lasing

**Figure 9:**
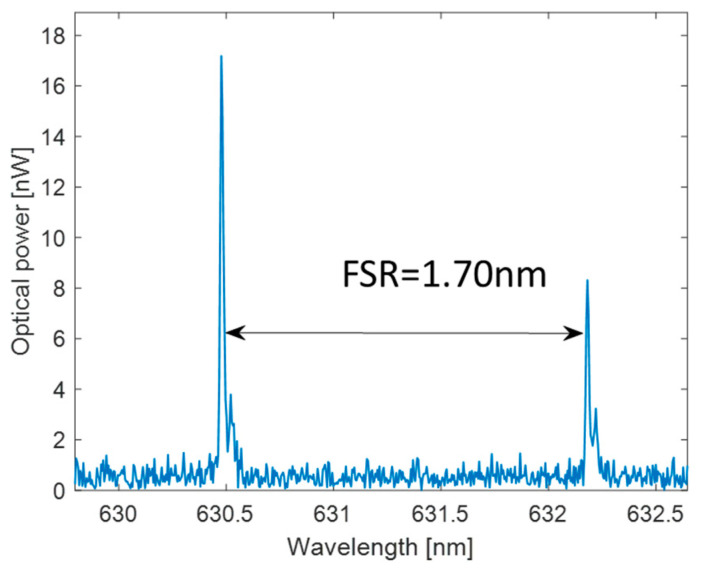
Measured spectrum transmitted by the Cavity B filled with water around 630 nm.

Figure 9 shows the measured transmission spectrum around 631 nm when the Cavity B was filled with water.

**Figure 10:**
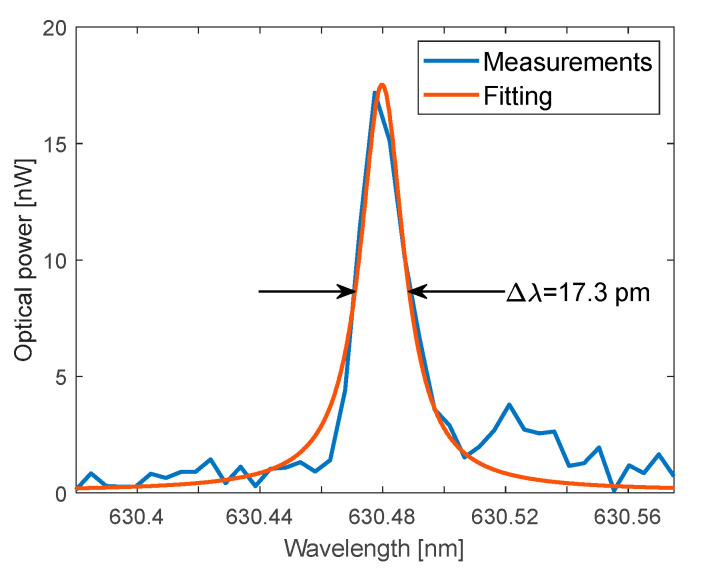
Lorentzian fitting of the FP resonance at λ_0_ ≈ 630 nm, Δλ = FWHM.

The measured FSR is 1.70 nm. Figure 10 shows a close-up view of the resonance peak at λ_0_~630.48 nm of Figure 9 for which a Lorentzian fit yields a resonance linewidth with a FWHM of Δλ = 17.3 pm, and the resulting quality factor is Q = λ_0_/Δλ = 3.64 × 10^4^. The cavity finesse is F = 98.

**Figure 11:**
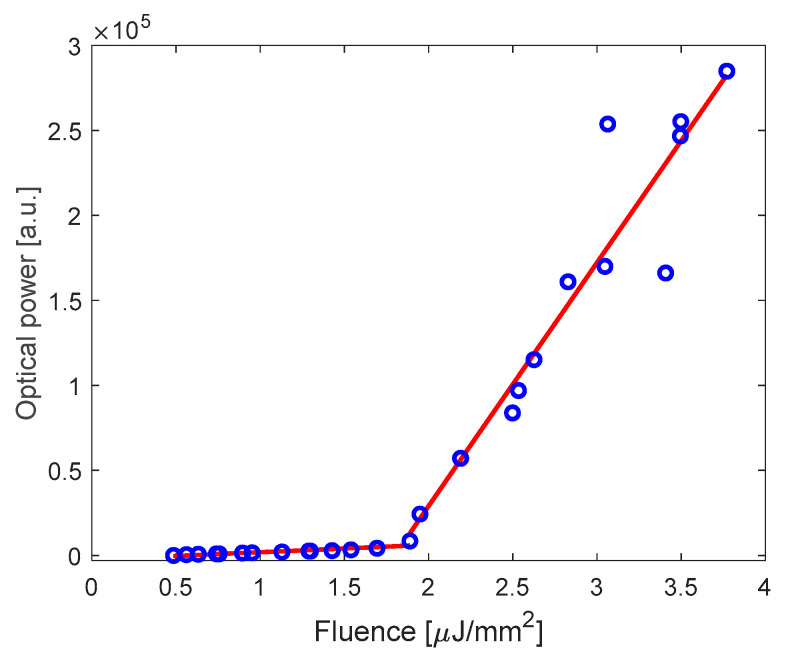
Spectrally integrated laser emission from 620 nm to 680 nm as a function of pump fluence. Solid lines are the linear fits, showing a lasing threshold of 1.83 μJ/mm^2^.

Figure 11 presents the spectrally integrated emission power across the 620–680 nm band as a function of incident pump fluence, showing characteristic nonlinear behavior that transitions from spontaneous to stimulated emission. Linear regression analysis of the sub-threshold and above-threshold regions reveals a distinct lasing threshold at 1.83 μJ/mm^2^. The threshold value is determined by the intersection point of linear fits to the spontaneous and amplified emission regimes, which defines the threshold condition.

The stability of the FP laser output is approximately 20%, which can be primarily attributed to two factors. First, the inherent fluctuations of the pump laser, which have a manufactured specified stability of about 8.5%, contribute directly to variations in the emitted power. Second, the presence of multiple longitudinal modes within the FP cavity leads to mode competition and interference effects, further influencing the output stability.

Remarkably, the measured threshold value is comparable to that of planar Fabry-Pérot cavities integrating high-performance DBR mirrors [22,24], despite our cavity being fabricated using polymeric mirrors and assembled using low-cost, simple polymer-based fabrication techniques.

**Figure 12:**
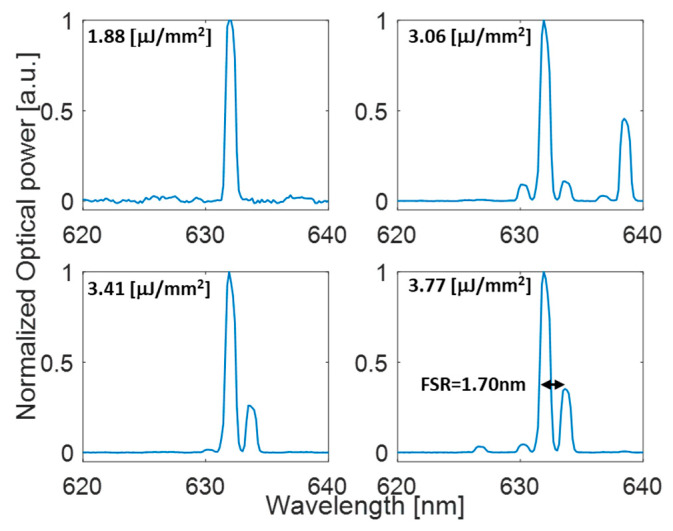
Lasing spectra emitted from FP cavity filled with 1 mM Rb solution in water.

Figure 12 shows the laser emission spectra of the FP cavity above threshold at four different pump fluences with resonance peaks centered at λ_0_ = 632 nm. The FSR of the cavity is 1.7 nm, in good agreement with transmission characterization.

## 4. Discussion

The experimental results reported in the previous sections demonstrate the possibility to develop high performance optical resonators with low-cost polymer materials.

As concerning sensing applications, the optical characterization reveals that the Cavity A exhibits a much higher Q-factor and Finesse compared to Cavity B, with a sensitivity. This performance difference is due primarily to DF2 higher reflectivity (R > 99%, 350–900 m). The DF2 mirrors 600-layer PEN/PMMA stack provides more effective interference conditions compared to DFB 250-layer PET/PMMA structure (R > 96%, 570–700 nm), resulting in sharper resonances.

To evaluate the sensing performance of the proposed device against other resonators, we calculate a figure of merit (FOM) of the sensor, defined as the sensitivity normalized to the FWHM of the resonance, i.e., FOM = S/FWHM.

For Cavity A we obtain FOM = 5.89 × 10^4^, with an improvement of a factor 4.2 compared to Cavity B and which represents one of the highest FOM achieved experimentally in the literature [19,25]. Furthermore, its extended reflectance range and high finesse allow detection of large refractive index changes or analyte-induced spectral shifts across a wide spectral window, enhancing its versatility as a platform for broadband or multi-analyte detection.

As concerning lasing applications, the spectral properties of the DFB mirrors of Cavity B, which exhibit a narrower reflectance band (570–700 nm), enable highly efficient optical pumping while simultaneously providing effective confinement of the fluorescence emission. This trade-off between Q-factor and cavity spectral selectivity demonstrates how cavity design must be optimized for specific applications. The achieved laser threshold of 1.83 μJ/mm^2^ is comparable with optofluidic lasers reported in the literature based on P-P FP [24] but realized with more complex and expensive fabrication process. In a comparison analysis of laser performances, it is also important to consider the choice of gain medium when evaluating lasing thresholds. In our experiments, Rhodamine B was used in aqueous solution, where its quantum yield is relatively low (∼0.23 at 1 mM) [26]. For comparison, other dyes such as Rhodamine 6G in ethanol offer significantly higher quantum yields (∼0.9), often used in the literature for low-threshold laser demonstrations. The employment in the future of water-soluble dyes with absorption/emission spectra similar to RhB, such as Atto 565 and Alexa Fluor 546, which exhibit quantum yields of 0.92 and 0.79, respectively, suggests that our lasing threshold values can be further improved under more favorable gain conditions for biosensing applications.

The proposed design of Cavity A and B fully implements a flow-through approach, with integrated microfluidics for sample handling. The fabrication process is simple, relying on lamination and micromachining steps that ensure good control over cavity dimensions. At the same time, the bonding strategy provides mechanical robustness and a leak-tight seal, allowing repeated fluid injection without compromising device integrity.

These results highlight the tunability and versatility of the proposed polymer-based P-P FP resonators. This adaptability, combined with ease of integration into microfluidic systems, make these devices as a promising platform for next-generation photonic sensors and microlasers.

## 5. Conclusions

This study demonstrates the feasibility and effectiveness of fully polymer-based optical microcavities for both refractive index sensing and lasing applications. Cavity A, utilizing high-reflectance polymeric mirrors, achieved a high Q-factor of Q = 2.15 × 10^5^ and fine spectral resolution, establishing its suitability for high-sensitivity label-free detection in microfluidic environments. Cavity B, though having a lower Q-factor, was successfully implemented as a dye-based laser cavity, enabled by the DFB mirrors selective reflectance band that matched the gain medium. The low lasing threshold of 1.83 μJ/mm^2^ supports the potential of these low-cost, scalable devices as integrated optical sensors.

## Data Availability

The raw data supporting the conclusions of this article will be made available by the authors on request.
